# Supplementary material for: Long-Term Effectiveness and Sustainability of Integrating Peer-Assisted Ultrasound Courses into Medical School—A Prospective Study
Source: Tomography. 2023 Jul 4;9(4):1315–28. doi: 10.3390/tomography9040104 (PMC10366829; doi:10.3390/tomography9040104)

Supplement Figure S1 Flow chart diagram of the study population (according to CONSORT); complete handling of the written tests and participation in the practical exam were the defined inclusion criteria

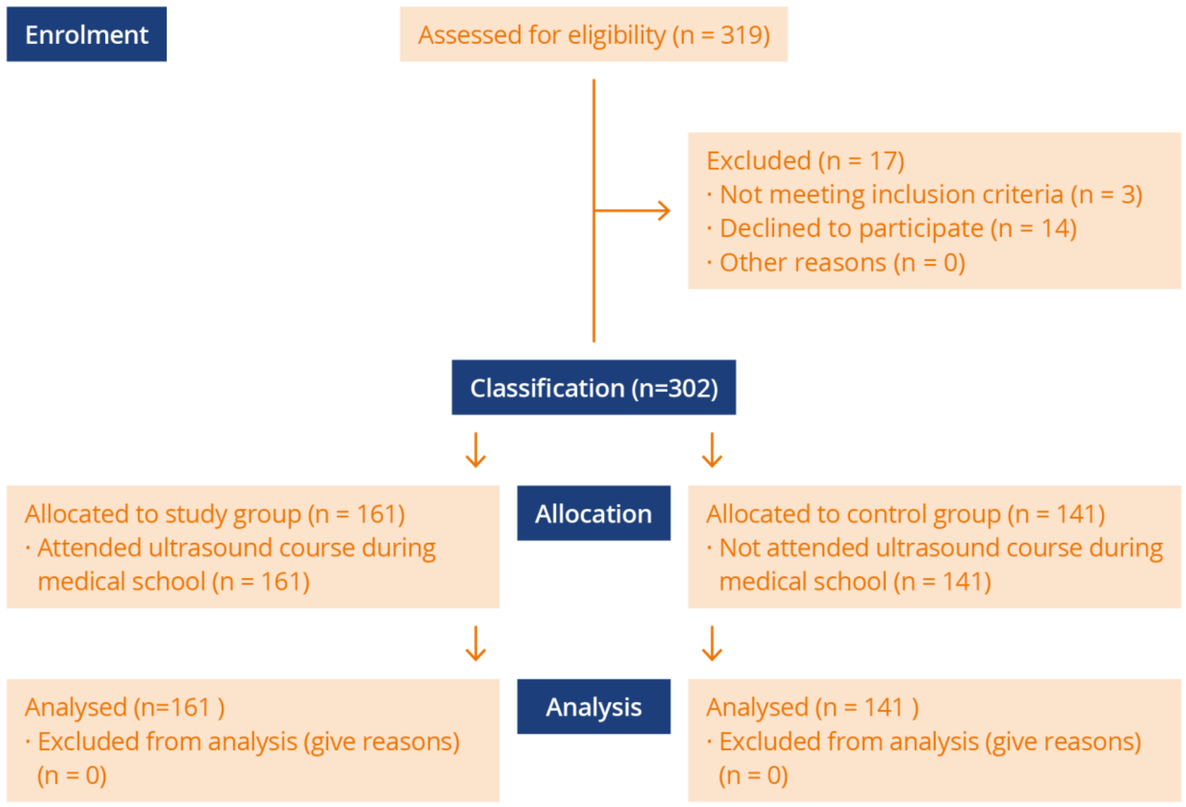

Supplement Figure S2 Theory test to check pathology understanding

Theory Questions Ultrasound Pathology

What tentative diagnosis comes to your mind when you look at the following ultrasound images?

a) Diagnosis:

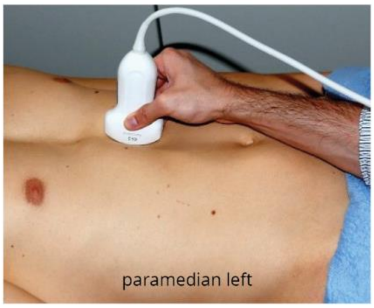

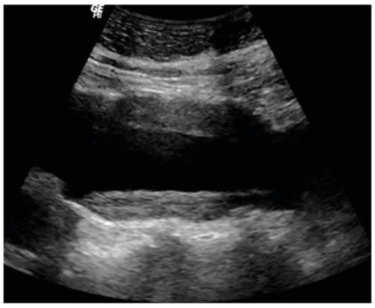

b) Diagnosis:

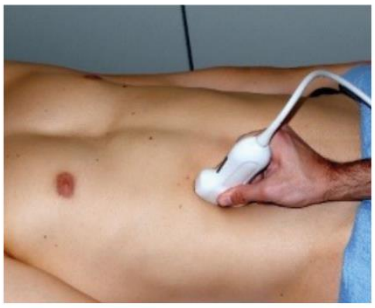

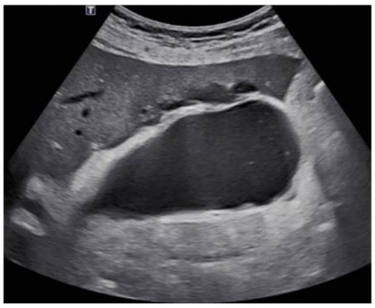

c) Diagnosis:

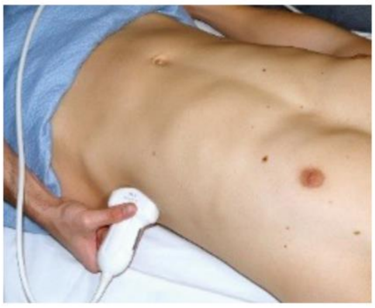

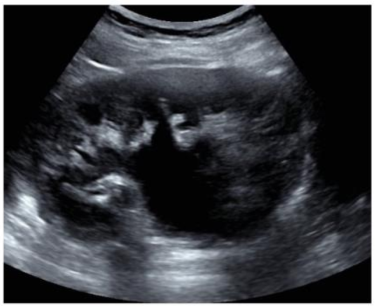

d) Diagnosis:

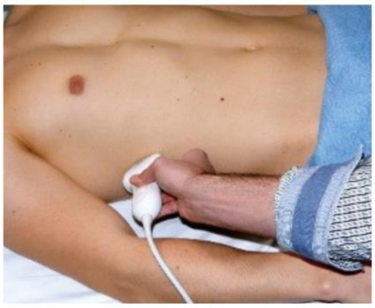

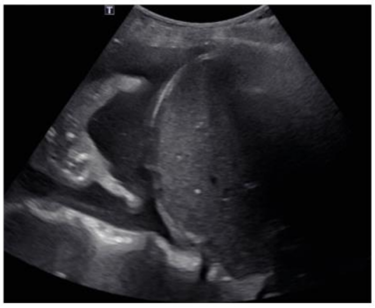

Supplementary 3: Examination task (lung, pleural effusions)

<u>**Case vignette:**</u> You are a physician in the emergency department and confronted with a patient (with a known tumour) who has been complaining of increasing shortness of breath for several days.

1) Please perform an ultrasound examination of the lungs and evaluate whether pleural effusions are a possible cause of the complaints.
2) Freeze the image and show the costodiaphragmatic recess, the diaphragm, and the liver.

Opening the dialogue

**Establishing a relationship (overall impression)**	
welcoming the patient , introduction , asking for the patient's name , or condition	☐2
	☐1
	☐0
**Establishing a good structure (overall impression)**	
names the occasion , asks about previous examination experiences , names the procedure , asks for permission for the examination	☐2
	☐1
	☐0

Handling the transducer

**Orientation**	
Correct, or immediately self-checked based on image movement, or by disconnecting the cranial part	☐2
Corrects after initial problems, or after instruction by the examiner	☐1
Finds correct orientation with manual help only	☐0
**Positioning**	
Correct, or immediately moved independently from another section	☐2
Corrects after initial difficulties, or after instruction by the examiner	☐1
Finds correct position with manual help only	☐0
**Connection**	
Connects transducer well, maintains pressure continuously (stabilizes transducer with fingers)	☐2
Corrects after initial problems, or following the examiner's instructions	☐1
Pressure not at all sufficient, half of the transducer in the air or uncontrolled pressure on rib	☐0
**Adequate adjustment of depth**	
Adjusts depth adequately and independently	☐2
Corrects after initial difficulties, or after instruction by the examiner	☐1
No adequate depth adjustment even under the examiner's instruction	☐0

**Patient guidance**	
Correct: "Please take a deep breath and hold it"	☐4
Incomplete, initial difficulty, or instruction by the examiner	☐2
Incomplete even after being reminded, or forgets several times to ask the patient to inhale	☐0
Prompts/Invites/Requests the patient to continue breathing	☐2

Examination

**Patient guidance**	
Complete on both sides	☐**10**
Complete on both sides, inadequate pace	☐**8**
Not complete on both sides, or complete on one side only	☐**6**
Only one side poorly, or dependent on the examiner´s help	☐**2**
Adequate scanning not possible	☐ **0**

Image explanation

**Shows and names the image correctly**				
Recess (1), Diaphragm (1), Liver (1)	☐**3**	☐**2**	☐**1**	☐**0**

Overall performance

Overall confident	☐**8**	☐**7**	☐**6**	☐**5**	☐**4**	☐**3**	☐**2**	☐**1**	*significant deficit*

Communication during the (physical) examination

**Consideration towards the patient (overall impression)**	
Empathetic communication , respects privacy , pays attention to nonverbal and paraverbal communication	☐**2**<br>☐**1**<br>☐**0**
**Result orientation of communication (overall impression)**	
Explains own actions , explains necessity of cooperation , gives clear instructions , pays attention to giving appropriate and understandable information	☐**2**<br>☐**1**<br>☐**0**

Theory

**What is the sonographic algorithm used for trauma patients in the emergency department called?**	
eFAST = extended focused assessment with sonography for trauma (1) , correct naming of the abbreviation (1)	☐**2**<br>☐**1**<br>☐**0**
**Please list ultrasound criteria that may be an indication of heart failure:**	
Progressive dilatation of the heart (1), B-lines (1), stasis in the inferior vena cava (1), pleural effusions (1), ascites (1)	☐**5**<br>☐**4**<br>☐**3**<br>☐**2**<br>☐**1**<br>☐**0**

Total score (max. 50):

____

Supplement Figure S3 Box plot diagram of subjective assessment of theoretical (a) and practical (b) competency

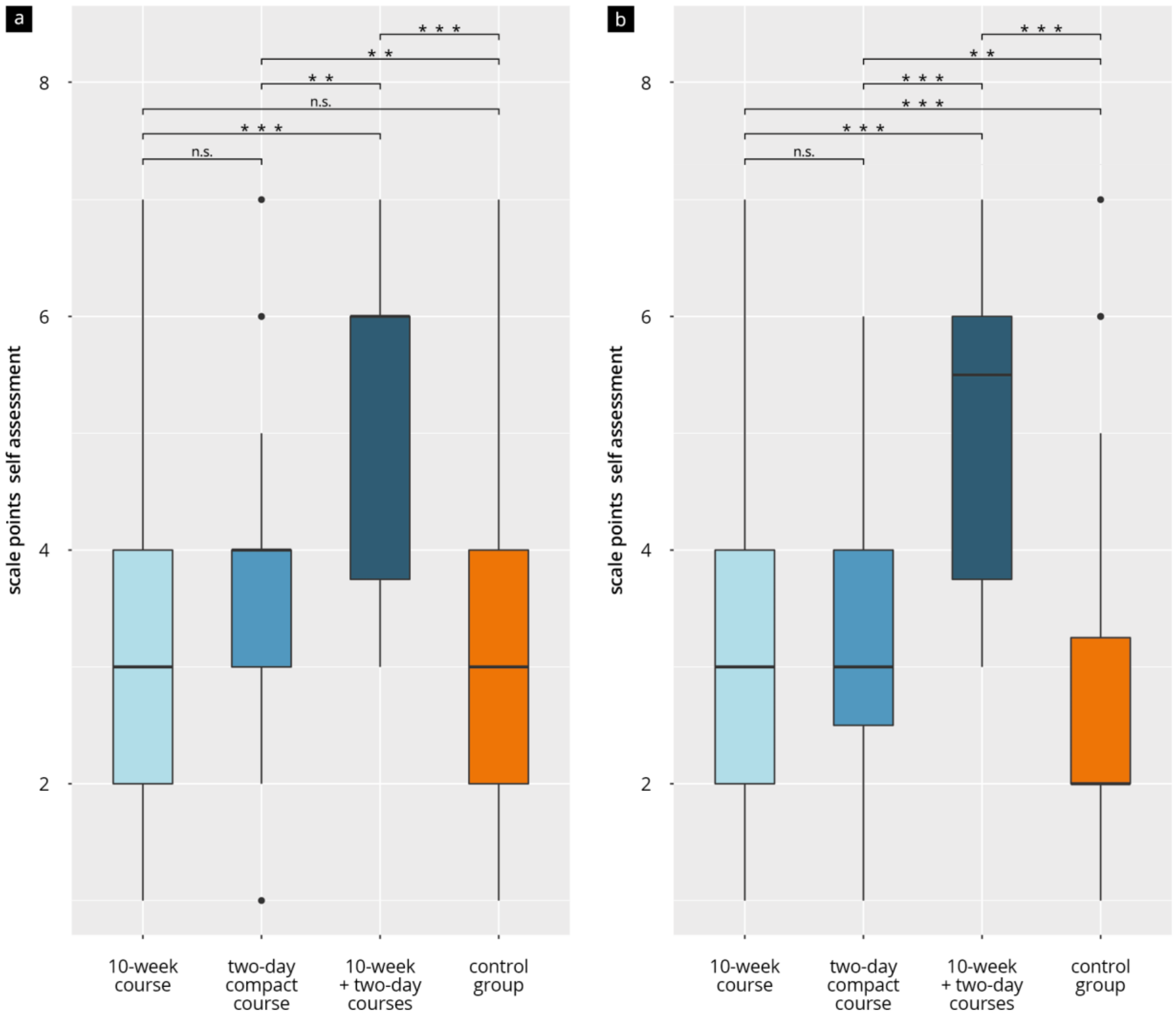

Supplement: Supplementary file 1 [file tomography-09-00104-s001.zip › tomography-2422648-supplementary.pdf]
